# Which is more effective in thawing frozen rooster sperm: varying temperature or duration?

**DOI:** 10.3389/fvets.2025.1534638

**Published:** 2025-01-22

**Authors:** Mustafa Yiğit Nizam, Murat Selçuk, Burcu Esin

**Affiliations:** ^1^Department of Reproduction and Artificial Insemination, Faculty of Veterinary Medicine, Dokuz Eylül University, İzmir, Türkiye; ^2^Department of Reproduction and Artificial Insemination, Faculty of Veterinary Medicine, Ondokuz Mayıs University, Samsun, Türkiye

**Keywords:** cryopreservation, post-thaw, rooster, sperm, dry thawing

## Abstract

**Introduction:**

Cryopreservation of poultry sperm is crucial for preserving genetic diversity and protecting endangered breeds. Rooster sperm is highly sensitive to cryopreservation due to its high polyunsaturated fatty acid content, making it prone to damage during freezing and thawing. This study evaluated the effects of thawing temperatures and storage conditions on sperm quality, including motility, morphology, and viability.

**Methods:**

Frozen rooster semen samples were thawed at 37°C for 30 seconds, 60°C for 5 seconds, or 72°C for 5 seconds and stored at 4°C for up to 48 hours. Sperm quality parameters, including motility, kinematic characteristics, abnormal morphology, and viability, were assessed at 0, 3, 6, 9, 12, 24, and 48 hours using a Computer-Assisted Semen Analyzer (CASA).

**Results:**

Post-thaw motility varied significantly between thawing temperatures at 24 and 48 hours (*p* < 0.05). Progressive and rapid progressive motility also differed significantly at 24 hours (*p* < 0.05). Sperm viability showed statistical differences across thawing groups at 24 and 48 hours (*p* < 0.05), while morphological abnormalities were significant at 12 and 48 hours (*p* < 0.05). Across all groups, sperm quality parameters varied significantly at each time point (*p* < 0.05).

**Discussion:**

Thawing at 37°C and storing at 4°C for up to 24 hours optimizes sperm motility and viability, minimizing cryodamage and ensuring functional preservation. This approach is effective for short-term storage and crucial for sustaining genetic diversity and fertility in poultry breeding programs.

## Introduction

Cryopreservation serves as a vital tool for preserving genetic diversity, particularly in economically significant and endangered species. While cryopreservation is a successful technique for preserving genetic material in various animal species, it is not widely adopted in the poultry industry due to a considerable reduction in the quality of stored sperm (1).

Compared to mammalian spermatozoa, rooster spermatozoa differ in having relatively less cytoplasm, mitochondria, and cytoplasmic antioxidants (2). However, the plasma membrane of rooster sperm is abundant in polyunsaturated fatty acids (PUFA), which are susceptible to oxidative stress (3) and vulnerable to damage during the freezing process (2).

Various studies have investigated different thawing temperatures and times for bull semen frozen in straws (4–9). These studies have consistently found that faster thawing methods lead to improved sperm motility and acrosomal integrity.

Slow thawing techniques, such as using ambient temperature, shirt pockets, or water baths at 20°C or lower, are generally discouraged as they negatively impact sperm survival, even if the sperm density is sufficient for fertilization. For bovine sperm frozen in straws, the recommended thawing method involves a water bath at 33–35°C for 30–40 s, regardless of the extender type and cooling rate, unless specific circumstances dictate otherwise (10, 11). However, some research has shown that raising the thawing temperature by 60–80°C can enhance sperm motility after thawing (10–12). Among the methods explored in recent publications, avian sperm is most commonly thawed in a water bath maintained at 37°C for 30 s, as this temperature is considered standard for achieving favorable results in terms of motility, viability, and overall post-thaw performance (13).

Despite advancements in thawing protocols for other species, there is limited research on the optimization of thawing procedures for rooster sperm. A recent study compared the effects of four different permeating cryoprotectants and two thawing temperatures (37 and 5°C) on post-thaw sperm motility and quality, concluding that ethylene glycol combined with thawing at 5°C significantly improved sperm motility and reduced apoptosis incidence (14). A comparative analysis of two thawing protocols (37°C for 30 s and 60°C for 5 s) revealed that the rapid thawing method at 60°C for 5 s significantly improved key sperm parameters, including total and progressive motility, mitochondrial functionality, and membrane integrity (15). Conventionally, thawed sperm is advised to be used immediately due to its rapid decline in motility post-thaw. However, the present study investigates the viability of rooster sperm stored at 4°C post-thaw and explores its potential for extended insemination periods.

This research addresses a critical knowledge gap in the optimization of thawing protocols for poultry sperm, emphasizing the need for a comprehensive understanding of how slow and rapid thawing methods affect sperm structure, viability, and functionality. Furthermore, it investigates the duration for which fertility parameters can be preserved when thawed rooster sperm is stored at 4°C, a suitable condition for short-term storage (16). This approach not only evaluates the effects of thawing temperature and duration but also examines changes in fertility parameters during 4°C storage. Additionally, it provides a practical framework for managing sperm that has been thawed due to unforeseen errors, such as leaks in liquid nitrogen tanks or the accidental thawing of sperm from an incorrect animal breed. The findings aim to contribute to the development of more efficient cryopreservation protocols tailored for both commercial breeding operations and conservation-focused applications.

## Materials and methods

The research was conducted in strict compliance with the guidelines outlined in the Ethics Committee decision (No. 2020/10) issued by the Poultry Research Institute under the Republic of Turkey Ministry of Agriculture and Forestry. Accordingly, the primary material utilized in this study comprised rooster semen straws cryopreserved using the protocol detailed below, as approved within the scope of this ethical framework.

### Management of roosters

Plymouth Rock roosters (*n* = 20), aged 49 weeks, were housed individually in cages under controlled environmental conditions, maintaining a photoperiod of 16 h light and 8 h dark. Semen samples were collected through dorso-abdominal massage, adhering to the standardized procedure established by Burrows and Quinn (17). To avert cold shock, samples were immediately placed in a heating block set to 37°C. Semen evaluation included assessments of volume, sperm motility, viability, and morphological integrity. Roosters with sperm motility of 90% or higher upon preliminary examination were included in the study. To minimize individual variability, semen samples collected from 20 roosters were pooled into a single collection container and subsequently diluted with *Beltsville Poultry Semen Extender* (BPSE). Extender Preparation and Cryopreservation.

The composition of *BPSE* is: Sodium glutamate (8.67 g/L), dipotassium phosphate trihydrate (12.70 g/L), sodium acetate (4.30 g/L), fructose (5 g/L), potassium citrate (0.64 g/L), n-tris-(hydroxymethyl)-methyl-1–2-aminoethanesulfonic acid (TES; 1.95 g/L), monopotassium phosphate (0.65 g/L), and magnesium chloride hexahydrate (0.34 g/L). Osmolarity and pH were adjusted to 333 mOsm/kg and 7.5, respectively. Glycerol was added at 5% (v/v) as a cryoprotectant.

Sperm samples diluted at a ratio of 1:30 were aspirated into 0.25 mL straws and sealed with polyvinyl alcohol powder. The total final sperm concentration was determined 770 × 10^6^ sperm per 0.25 mL straw. The straws were equilibrated at 4°C for 2 h. Following equilibration, they were frozen in liquid nitrogen vapor, positioned 4 cm above the liquid nitrogen surface in a cryobox for 7 min. Subsequently, the straws were immersed in liquid nitrogen for storage until examination.

### Thawing procedures

Frozen rooster semen contained in 0.25 mL straws was thawed using three distinct protocols: at 37°C for 30 s, at 60°C for 5 s, and at 72°C for 5 s in a water bath. Five straws were thawed for each protocol. Glycerol was not removed after thawing because its application in field conditions is not feasible, and our study was designed based on field conditions. Thawed sperm samples were transferred to 0.5 mL Eppendorf tubes and stored at 4°C.

Motility, kinematic parameters, viability, and morphological assessments were conducted immediately post-thaw (0 h) for each thawing protocol. The remaining semen in Eppendorf tubes was stored at 4°C, and spermatological evaluations were subsequently repeated at 3, 6, 9, 12, 24, and 48 h post-thaw.

### Motility and sperm kinematics

Total motility and progressive motility were examined with a CASA (Sperm Class Analyzer®, version 6.3.0.59, Microptic, Barcelona, Spain). The slide (Leja 20 μm) was placed onto a stage warmer set at 37°C. At least five microscopic fields and 500 spermatozoa were analyzed for each sample. For kinematic characteristics of sperm movement; straight-line velocity (VSL; μm/s), curvilinear velocity (VCL; μm/s), average path velocity (VAP; μm/s), amplitude of lateral head displacement (ALH; μm), linearity (LIN; VSL/VCL × 100), wobble (WOB; VAP / VCL × 100), straightness (STR; VSL/VAP × 100), and beat-cross frequency (BCF; Hz) were determined with the software system.

### Sperm morphology

Spermatozoa were morphologically evaluated by using Sperm Blue^®^ (Microptic, Barcelona, Spain). Ten milliliter of sperm samples were smeared across on a clear slide and allowed to air dry. After the samples dried, smears were stained in a Coplin-type jar with Sperm Blue Stain^®^ for 1 min. After preparation, slides were examined using the CASA system at 60x magnification. Head, midpiece, and tail abnormalities, as well as total spermatozoa abnormalities, were quantified as percentages (%).

### Sperm viability

The eosin-nigrosine stain was used to assess sperm viability as described by Raseona et al. (18). After staining, the slides were dried and covered with a cover slip before evaluation by using CASA at 60x magnification. The rates (%) of live (white sperm heads) and dead (pink sperm heads) sperm were determined by counting a total of 200 spermatozoa per each stained slide.

### Statistical analysis

To evaluate the effects of varying durations and temperatures on kinematic and morphological parameters, a factorial experimental design was employed. The primary effects of time and temperature, as well as their interaction effects, were analyzed and compared. General linear model with the Duncan multiple comparison test was utilized for assessing the main effects due to its efficiency in controlling Type I error and its ability to provide clear group separations. Interaction effects were further examined for significant differences between groups using the Tukey test, which is particularly effective in identifying pairwise differences while maintaining a balance between Type I and Type II error rates. Descriptive statistics were calculated to summarize the data.

The sample size for the study was determined through a preliminary power analysis to ensure sufficient statistical power (≥ 0.80) for detecting significant differences between groups with a significance level of 0.05. Randomization procedures were implemented to assign experimental units to different treatment groups, ensuring unbiased representation of conditions.

All analyses and computations in this study were performed using SAS statistical software (19), ensuring reproducibility and accuracy of the results.

## Results

The evaluation of motion parameters, as presented in Table 1. Statistically significant differences (*p* < 0.05) were noted among the 0, 3, 6, 9, 12, 24, and 48-h time points post-thaw across the three distinct thawing protocols. Notably, at 24 and 48 h following thawing at 37°C, motility values were 62.15 ± 8.37% and 37.95 ± 2.61%, respectively, representing the highest motility percentages compared with both alternative thawing protocols and time intervals.

**Table 1:** Spermatological parameter at different time and temperature after thawing.

	Parameter	Temperature (°C)	Storage time (h)							
			0	3	6	9	12	24	48	Linear
			$\bar{X}\pm S_{\bar{X}}$	$\bar{X}\pm S_{\bar{X}}$	$\bar{X}\pm S_{\bar{X}}$	$\bar{X}\pm S_{\bar{X}}$	$\bar{X}\pm S_{\bar{X}}$	$\bar{X}\pm S_{\bar{X}}$	$\bar{X}\pm S_{\bar{X}}$	
Motion parameters	Motility (%)	37	86.09 ± 1.01^a^	83.52 ± 1.95^a^	78.02 ± 1.59^ab^	75.83 ± 3.14^ab^	71.18 ± 2.11^b^	62.15 ± 8.37^bcx^	37.95 ± 2.61^dx^	*
		60	84.24 ± 1.12^a^	82.49 ± 0.78^ab^	78.30 ± 1.94^ab^	79.92 ± 1.92^ab^	71.63 ± 2.95^b^	46.43 ± 5.65^cy^	27.27 ± 7.62^dy^	*
		72	78.53 ± 2.89^a^	78.79 ± 1.38^a^	78.10 ± 1.28^a^	73.74 ± 1.96^a^	76.24 ± 2.43^a^	61.50 ± 3.73^bx^	16.73 ± 7.14^cz^	*
	Linear		NS	NS	NS	NS	NS	*	*	
	Progressive motility (%)	37	20.03 ± 1.40^a^	17.34 ± 1.81^ax^	16.95 ± 0.89^ax^	16.94 ± 1.80^ax^	11.68 ± 1.13^b^	13.28 ± 1.84^bx^	2.43 ± 0.84^c^	*
		60	17.36 ± 0.40^a^	15.34 ± 0.79^abx^	13.00 ± 1.03^bcy^	13.09 ± 0.77^bcy^	10.21 ± 0.77^c^	5.49 ± 1.47^dy^	2.33 ± 0.85^d^	*
		72	21.07 ± 1.81^a^	13.63 ± 1.49^by^	12.04 ± 0.91^by^	11.23 ± 0.52^by^	13.14 ± 1.26^b^	12.47 ± 1.73^bx^	2.30 ± 0.96^c^	*
	Linear		NS	*	*	*	NS	*	NS	
	Rapid progressive motility (%)	37	4.14 ± 0.4^a^	3.12 ± 0.39^b^	3.26 ± 0.23b^x^	3.23 ± 0.40^bx^	2.17 ± 0.23^c^	2.57 ± 0.36^bcx^	0.50 ± 0.21^d^	*
		60	3.37 ± 0.14^a^	2.83 ± 0.21^ab^	2.50 ± 0.19^bx^	2.38 ± 0.19^by^	1.85 ± 0.16^bc^	0.96 ± 0.26^dy^	0.19 ± 0.12^d^	*
		72	3.75 ± 0.4^a^	2.33 ± 0.29^b^	1.98 ± 0.25^by^	2.12 ± 0.23^by^	2.44 ± 0.21^b^	2.38 ± 0.43^bx^	0.20 ± 0.13^c^	*
	Linear		NS	NS	*	*	NS	*	NS	

*p < 0.05. NS, not significant. a, b, c, d: Values corresponding to each distinct letter in each row are statistically significant. x, y, z: Values corresponding to each distinct letter in each column are statistically significant.

Post-thaw assessments of progressive motility at the 3rd, 6th, 9th, and 24th hours demonstrated statistically significant variation (*p* < 0.05) across the different thawing protocols, indicating substantial procedural impacts on the maintenance of progressive motility over time. However, by the 48th hour, progressive motility values converged, showing no statistically significant differences across thawing treatments (*p* > 0.05), potentially reflecting the long-term equilibration of motility attributes under each protocol. In a similar trend, rapid progressive motility values were significantly different (*p* < 0.05) among thawing procedures at the 6th, 9th, and 24th hours, underscoring distinct effects of thawing methodology on the preservation of this motility parameter.

The analysis of kinematic parameters, detailed in Table 2, revealed pronounced alterations (*p* < 0.05) in VCL, VAP, VSL, ALH, and BCF among semen samples stored at 4°C up to 48 h following thawing. These parameters exhibited time-dependent sensitivity to thawing protocols, potentially influencing the kinematic behavior of spermatozoa across the post-thaw storage interval.

**Table 2:** Kinematic parameter values at different time and temperature after thawing.

	Parameter	Temperature (°C)	Storage time (h)							Linear
			0	3	6	9	12	24	48	
			$\bar{X}\pm S_{\bar{X}}$	$\bar{X}\pm S_{\bar{X}}$	$\bar{X}\pm S_{\bar{X}}$	$\bar{X}\pm S_{\bar{X}}$	$\bar{X}\pm S_{\bar{X}}$	$\bar{X}\pm S_{\bar{X}}$	$\bar{X}\pm S_{\bar{X}}$	
Kinematics	VCL (μm/s)	37	57.66 ± 2.74^a^	53.30 ± 3.18^a^	56.93 ± 3.10^ax^	57.15 ± 3.28^ax^	43.79 ± 2.43^b^	50.51 ± 2.61a^bx^	26.60 ± 2.88^c^	*
		60	52.83 ± 1.02^a^	50.15 ± 1.77^a^	45.09 ± 1.74^aby^	45.46 ± 2.09^aby^	40.43 ± 0.96^bc^	34.84 ± 3.43^cy^	22.39 ± 6.06^d^	*
		72	65.58 ± 3.90^a^	46.43 ± 2.81^bc^	43.99 ± 1.79^bcy^	42.08 ± 1.31^by^	46.06 ± 2.35^bc^	52.37 ± 3.25^cx^	19.92 ± 8.28^d^	*
	Linear		NS	NS	*	*	NS	*	NS	
	VAP (μm/s)	37	26.74 ± 1.57^a^	24.63 ± 1.58^ab^	26.43 ± 1.19^ax^	25.96 ± 1.65^ax^	19.81 ± 1.07^b^	24.47 ± 1.71^abx^	10.25 ± 1.84^c^	*
		60	23.92 ± 0.40^a^	23.36 ± 0.81^a^	21.43 ± 0.93^aby^	20.72 ± 0.70^abcy^	17.84 ± 0.56^b^	16.26 ± 2.05^bcy^	10.02 ± 3.09^d^	*
		72	30.61 ± 2.39^a^	20.47 ± 1.69^b^	20.08 ± 0.99^by^	19.57 ± 0.81^by^	21.30 ± 1.21^b^	23.96 ± 1.78^bx^	8.77 ± 3.96^c^	*
	Linear		NS	NS	*	*	NS	*	NS	
	VSL (μm/s)	37	13.79 ± 0.85^a^	12.67 ± 0.86^ab^	13.82 ± 0.49^ax^	13.12 ± 0.91^ab^	10.59 ± 0.53^b^	13.62 ± 0.73^ax^	5.25 ± 1.22^c^	*
		60	12.50 ± 0.33^a^	12.25 ± 0.29^a^	11.50 ± 0.60^abxy^	10.73 ± 0.34^abc^	9.20 ± 0.41^b^	8.68 ± 1.12^bcy^	5.26 ± 1.85^d^	*
		72	15.71 ± 1.35^a^	10.27 ± 1.02^b^	10.46 ± 0.73^by^	10.61 ± 0.58^b^	11.34 ± 0.67^b^	12.46 ± 0.95^bx^	5.22 ± 2.35^c^	*
	Linear		NS	NS	*	NS	NS	*	NS	
	STR (%)	37	37.24 ± 0.77	36.66 ± 0.62	37.42 ± 1.73	37.14 ± 0.93	38.01 ± 0.88	40.04 ± 1.62	38.90 ± 1.33^x^	NS
		60	37.18 ± 0.75	39.48 ± 1.31	40.08 ± 1.79	37.89 ± 1.02	36.65 ± 0.72	39.73 ± 0.36	35.38 ± 9.48^x^	NS
		72	38.23 ± 1.17^a^	35.62 ± 1.05^a^	38.68 ± 2.85^a^	39.52 ± 0.55^a^	37.72 ± 1.14^a^	37.01 ± 0.41^a^	24.95 ± 10.29^by^	*
	Linear		NS	NS	NS	NS	NS	NS	*	
	LIN (%)	37	16.41 ± 0.71	16.71 ± 0.43	17.55 ± 0.73	15.38 ± 0.59	16.42 ± 0.70	20.36 ± 1.57	13.30 ± 1.35	NS
		60	16.15 ± 0.54	17.84 ± 0.98	18.95 ± 1.72	16.20 ± 0.52	14.70 ± 0.89	18.20 ± 0.86	15.53 ± 6.12	NS
		72	17.46 ± 1.19^a^	14.78 ± 1.28^ab^	17.08 ± 2.67^a^	17.62 ± 0.76^a^	16.49 ± 0.74^ab^	15.88 ± 0.52^ab^	10.76 ± 5.13^b^	*
	Linear		NS	NS	NS	NS	NS	NS	NS	
	WOB (%)	37	37.44 ± 0.85	36.91 ± 0.63	37.54 ± 1.03	35.68 ± 0.56	36.08 ± 0.89	41.30 ± 1.56	30.40 ± 1.54	NS
		60	36.49 ± 0.47	37.89 ± 0.60	39.48 ± 1.47	36.70 ± 0.87	34.48 ± 1.04	38.91 ± 1.53	30.33 ± 8.96	NS
		72	38.19 ± 1.49^a^	35.35 ± 1.44^a^	36.83 ± 2.67^a^	37.37 ± 0.80^a^	36.93 ± 1.03^a^	35.88 ± 0.66^a^	21.96 ± 9.45^b^	*
	Linear		NS	NS	NS	NS	NS	NS	NS	
	ALH (μm)	37	1.69 ± 0.07^a^	1.59 ± 0.08^ab^	1.68 ± 0.07^ax^	1.68 ± 0.08^ax^	1.33 ± 0.06^b^	1.53 ± 0.09^abx^	0.85 ± 0.07^c^	*
		60	1.58 ± 0.02^a^	1.50 ± 0.04^ab^	1.37 ± 0.05^abcy^	1.36 ± 0.05^abcy^	1.25 ± 0.02^bc^	1.13 ± 0.10^cy^	0.73 ± 0.19^d^	*
		72	1.92 ± 0.11^a^	1.40 ± 0.07^b^	1.34 ± 0.04^by^	1.31 ± 0.03^by^	1.40 ± 0.06^b^	1.56 ± 0.07^bx^	0.62 ± 0.26^c^	*
	Linear		NS	NS	*	*	NS	*	NS	
	BCF (Hz)	37	3.54 ± 0.14^a^	3.44 ± 0.17^a^	3.35 ± 0.13^a^	3.20 ± 0.13^a^	2.86 ± 0.17^a^	3.09 ± 0.17^ax^	1.65 ± 0.15^b^	*
		60	3.11 ± 0.06^ab^	3.35 ± 0.13^a^	3.52 ± 0.30^a^	2.96 ± 0.07^ab^	2.56 ± 0.07^bc^	2.17 ± 0.13^cy^	1.97 ± 0.64^c^	*
		72	3.41 ± 0.19^a^	2.82 ± 0.12^a^	3.09 ± 0.21^a^	2.86 ± 0.13^a^	2.98 ± 0.17^a^	2.82 ± 0.16^axy^	1.36 ± 0.55^b^	*
	Linear		NS	NS	NS	NS	NS	*	NS	

*p < 0.05. NS = not significant. a, b, c, d: Values corresponding to each distinct letter in each row are statistically significant. x, y: Values corresponding to each distinct letter in each column are statistically significant.

Table 3 presents findings related to the viability and morphological integrity of spermatozoa, where viability outcomes at 24 and 48 h varied significantly (*p* < 0.05) among the three thawing procedures, signifying that certain protocols may more effectively support cellular survival over prolonged post-thaw periods. Morphological assessments also showed considerable variation, with statistically significant differences (*p* < 0.05) in head abnormalities observed at 0, 12, and 48 h, in midpiece abnormalities at 0, 3, 6, 12, 24, and 48 h, and in tail morphology at 0, 12, and 24 h. Notably, total abnormal spermatozoa counts diverged markedly (*p* < 0.05) at 0, 12, and 48 h, further indicating that morphological stability was contingent on both thawing protocol and storage duration.

**Table 3:** Statistical results of sperm morphology and viability rate after thawing.

	Parameter	Temperature (°C)	Storage time (h)							Linear
			0	3	6	9	12	24	48	
			$\bar{X}\pm S_{\bar{X}}$	$\bar{X}\pm S_{\bar{X}}$	$\bar{X}\pm S_{\bar{X}}$	$\bar{X}\pm S_{\bar{X}}$	$\bar{X}\pm S_{\bar{X}}$	$\bar{X}\pm S_{\bar{X}}$	$\bar{X}\pm S_{\bar{X}}$	
Morphology	Viability (%)	37	86.60 ± 0.74^a^	85.60 ± 2.20^ab^	81.00 ± 2.12^ab^	74.80 ± 2.24^bc^	68.60 ± 3.18^cd^	61.60 ± 9.10^dx^	37.00 ± 4.18^ex^	*
		60	85.60 ± 1.63^a^	81.60 ± 0.92^ab^	77.60 ± 2.50^ab^	80.60 ± 1.93^ab^	71.60 ± 3.48^b^	46.00 ± 7.86^cy^	28.60 ± 8.00^dx^	*
		72	80.40 ± 4.38^a^	78.60 ± 1.32^a^	81.20 ± 2.03^a^	74.60 ± 1.50^a^	76.80 ± 2.43^a^	62.80 ± 3.87^bx^	14.00 ± 6.09^cy^	*
	Linear		NS	NS	NS	NS	NS	*	*	
	Head (%)	37	3.40 ± 0.87^ax^	4.80 ± 0.96^ab^	5.00 ± 0.31^ab^	6.40 ± 0.50^bc^	7.20 ± 0.66^cx^	8.40 ± 0.87^c^	11.20 ± 0.80^dx^	*
		60	3.60 ± 0.67^ax^	3.40 ± 0.67^a^	4.00 ± 0.31^a^	7.00 ± 0.70^b^	10.60 ± 0.92^cy^	10.40 ± 0.74^c^	12.60 ± 0.50^dx^	*
		72	6.00 ± 0.83^ay^	3.80 ± 0.66^b^	6.60 ± 0.6^7a^	7.20 ± 0.73^a^	6.40 ± 0.67^ax^	6.80 ± 0.86^a^	16.40 ± 0.74^cy^	*
	Linear		*	NS	NS	NS	*	NS	*	
	Midpiece (%)	37	8.40 ± 1.74^ax^	18.60 ± 1.16^bdx^	14.40 ± 1.07^cx^	16.40 ± 1.07^bcd^	17.60 ± 0.50^bcdx^	18.60 ± 0.92^dx^	18.40 ± 0.74^dx^	*
		60	9.40 ± 1.53^ax^	16.80 ± 1.06^bcdxy^	15.00 ± 0.54^bcdxy^	14.00 ± 0.70^bd^	16.80 ± 0.73^bcdx^	17.80 ± 0.86^cdxy^	16.00 ± 0.70^dx^	*
		72	18.00 ± 3.34^acy^	13.80 ± 1.15^bdey^	17.80 ± 0.80^cey^	17.20 ± 0.86^ce^	13.20 ± 0.80^dey^	14.60 ± 0.60^ey^	23.80 ± 0.86^fy^	*
	Linear		*	*	*	NS	*	*	*	
	Tail (%)	37	32.40 ± 1.63^ax^	27.00 ± 0.89^b^	38.80 ± 0.86^c^	41.80 ± 1.35^cd^	43.20 ± 1.39^cdex^	45.00 ± 1.18^de^	47.60 ± 1.28^ex^	*
		60	31.20 ± 1.85^ax^	27.40 ± 1.16^a^	39.00 ± 0.89^b^	43.20 ± 1.06^bd^	49.20 ± 0.86^cdy^	46.20 ± 0.58^d^	58.80 ± 0.73^ey^	*
		72	41.60 ± 5.30^acdy^	30.20 ± 1.01^b^	40.20 ± 0.86^c^	45.20 ± 0.86^de^	47.60 ± 1.20^ey^	48.00 ± 0.70^e^	49.00 ± 0.70^ex^	*
	Linear		*	NS	NS	NS	*	NS	*	
	Total Abnormal (%)	37	44.20 ± 3.72^ax^	50.40 ± 2.42^a^	58.20 ± 1.49^b^	64.60 ± 2.58^bc^	68.00 ± 2.07^cdx^	72.00 ± 2.46^de^	77.20 ± 2.47^ex^	*
		60	44.20 ± 1.62^ax^	47.60 ± 0.92^a^	58.00 ± 1.64^b^	64.20 ± 1.49^b^	76.60 ± 1.80^cy^	74.40 ± 2.11^c^	87.40 ± 1.16^dy^	*
		72	65.60 ± 6.91^acy^	47.80 ± 1.98^b^	64.60 ± 2.03^c^	69.60 ± 2.08^c^	67.20 ± 2.61^cx^	69.40 ± 1.46^c^	89.20 ± 1.65^dy^	*
	Linear		*	NS	NS	NS	*	NS	*	

*p < 0.05.NS = Not Significant. a, b, c, d, e, f: Values corresponding to each distinct letter in each row are statistically significant. x, y: Values corresponding to each distinct letter in each column are statistically significant.

## Discussion

Cryopreservation imposes both chemical and physical stresses on the sperm membrane, adversely affecting motility, viability, and membrane integrity (20). Consequently, the thawing process is critically important, as it significantly influences post-thaw sperm survival, paralleling the importance of the freezing process itself (7). Enhanced viability post-thaw is directly associated with improved sperm fertility outcomes (21). In the current study, viability remained statistically similar at 0, 3, 6, 9, and 12 h following storage at 4°C post-thaw, yet a marked decline was observed at 24 and 48 h (*p* < 0.05). These findings suggest that rooster spermatozoa stored at 4°C maintained their viability effectively for up to 12 h post-thaw, with limited impact from the different thawing protocols applied.

Purdy (22) highlighted the importance of the thawing phase, particularly the transition from −196°C to 4°C, due to the risk of recrystallization affecting sperm integrity. In the current study, the selected thawing rates did not compromise sperm motility within the initial 12-h period post-thaw. Beyond the immediate findings, this study emphasizes the critical role of tailored cryopreservation techniques in sustaining genetic diversity and improving fertility outcomes. By bridging the gap between theoretical optimization and practical application, the results contribute significantly to advancing poultry reproductive technologies.

Thawing plays a critical role in preserving sperm quality and viability, although it has been underexplored by researchers (23). Generally, a rapid thawing rate is essential to minimize the risk of recrystallization. The findings of this study demonstrate that elevated thawing temperatures significantly enhance sperm metabolism and membrane integrity. This method effectively reduces ice crystal formation and recrystallization, while potentially enhancing enzyme activity (24). Additionally, this rapid thawing approach reduces the exposure duration to concentrated solutes and glycerol, and it expedites the restoration of extracellular and intracellular balance, unlike slower thawing processes (25). The transition from −196 to 4°C is particularly critical due to the high likelihood of recrystallization (22). In terms of thawing rates, a higher temperature (72°C for 5 s) outperformed a lower one (37°C for 30 s) in maintaining sperm quality, with notable improvements in both total and progressive motility for the faster thawing temperature (72°C for 5 s).

The findings of Salih et al. (15) indicate motility rates of 57.92 and 60.58% for rooster semen thawed at 37°C and 60°C, respectively, with progressive motility values of 22.86 and 24.76%. In comparison, Fattah et al. (26) observed a motility rate of 52.7% and a progressive motility rate of 19.4% for semen thawed at 37°C. In contrast to these studies, the present research reports considerably higher motility rates of 86.09, 84.24, and 78.53% for thawing temperatures of 37, 60, and 72°C, respectively, with corresponding progressive motility values of 20.03, 17.36, and 21.07%. By the 6th hour post-thaw, motility rates for the 37, 60, and 72°C conditions were recorded as 78.02, 78.30, and 78.10%, respectively.

Of particular interest, the rapid thawing temperature of 72°C for a duration of 5 s initially produced the highest progressive motility but showed a substantial decline by the 6th hour, suggesting that elevated initial thawing temperatures may have short-term benefits for progressive motility that diminish over time. Generally, the motility rates observed in this study surpass those documented by Salih et al. (15) and Fattah et al. (26), while the progressive motility values for semen thawed at 37°C closely approximate those previously reported, indicating potential consistencies in progressive motility outcomes across different studies.

The application of CASA has gained significant traction for evaluating sperm kinematic parameters, offering a more accurate and objective assessment of sperm motility than traditional subjective methods (27). Studies have highlighted correlations between fertility and specific sperm kinematic parameters, noting that values such as VCL, VSL, and VAP may serve as reliable predictors of *in vivo* fertility outcomes (28, 29). In the present study, however, the analysis revealed no statistically significant differences among the three thawing temperatures (37, 60, and 72°C) with respect to these velocity parameters at 0, 3, 12, and 24 h post-thawing. This outcome suggests that variations in thawing temperature may not markedly influence these specific kinematic indicators over time. These insights not only pave the way for enhanced cryopreservation protocols but also establish a foundation for future studies exploring the molecular and biochemical mechanisms underlying sperm resilience during the thawing process. Such investigations could further refine our understanding of how cryopreservation strategies can be optimized to balance long-term storage efficiency and post-thaw functionality, ultimately supporting both commercial and conservation-focused breeding programs.

Nur et al. (7) have shown that the method of thawing can influence morphological damage in spermatozoa, a finding echoed in more recent studies. For instance, Najafi et al. (3) reported rooster semen viability rates of 58.46% after thawing at 37°C, alongside a 20.91% rate of total abnormal spermatozoa. Similarly, Salih et al. (15) documented sperm viability rates of 60.80 and 63.21% after thawing at 37°C and 60°C, respectively, with total abnormal spermatozoa rates of 17.11 and 16.19%. In contrast, the present study demonstrated higher sperm viability and abnormality rates post-thaw, with viability values of 86.60 and 85.60% for semen thawed at 37°C and 60°C, respectively, and a slightly lower rate of 80.40% at 72°C. The total abnormal spermatozoa rates were notably higher at 44.20% for 37°C and 60°C, and 65.60% for 72°C.

Interestingly, while thawing at 37°C produced the highest initial sperm viability, by the 24th hour, semen thawed at 72°C showed the highest sustained viability. At the 48-h mark, however, the highest viability rate was again observed in semen thawed at 37°C. Regarding morphological integrity, the lowest abnormal spermatozoa rates were recorded for semen thawed at 37 and 60°C. Nonetheless, by the 12th and 24th hours post-thaw, the 72°C group showed the least morphological damage, suggesting a potential protective effect of rapid thawing against structural abnormalities over time. Overall, these findings align with prior studies, including Nur et al. (7) and Senger (12), reinforcing the significant impact of thawing techniques on sperm morphology.

## Conclusion

This study demonstrates that thawing at 37°C for 30 s consistently achieves superior preservation of motility and viability over extended post-thaw periods, establishing it as the optimal choice for long-term storage and practical insemination scenarios. Conversely, the rapid thawing method at 72°C for 5 s demonstrated significant short-term advantages, particularly in reducing ice recrystallization and enhancing immediate sperm functionality. These findings highlight the flexibility of thawing strategies to meet varying practical and operational needs in poultry breeding programs.

Ultimately, this research provides a practical framework for enhancing the efficiency of poultry breeding programs by implementing optimized cryopreservation protocols tailored to specific operational needs, such as short-term insemination or rapid functionality recovery.

## Data Availability

The original contributions presented in the study are included in the article/supplementary material, further inquiries can be directed to the corresponding author.
